# Metric to quantify white matter damage on brain magnetic resonance images

**DOI:** 10.1007/s00234-017-1892-1

**Published:** 2017-08-16

**Authors:** Maria Del C. Valdés Hernández, Francesca M. Chappell, Susana Muñoz Maniega, David Alexander Dickie, Natalie A. Royle, Zoe Morris, Devasuda Anblagan, Eleni Sakka, Paul A. Armitage, Mark E. Bastin, Ian J. Deary, Joanna M. Wardlaw

**Affiliations:** 1 Department of Neuroimaging Sciences, Centre for Clinical Brain Sciences, University of Edinburgh, Chancellor’s Building, 49 Little France Crescent, Edinburgh, EH16 4SB UK; 2 Centre for Cognitive Ageing and Cognitive Epidemiology, University of Edinburgh, Edinburgh, UK; 3 Edinburgh Dementia Research Centre, UK Dementia Research Institute, London, UK; 4 Department of Cardiovascular Sciences, University of Sheffield, Sheffield, UK; 5 Department of Psychology, University of Edinburgh, Edinburgh, UK

**Keywords:** MRI, Brain, Cerebrovascular disorders, Leukoencephalopathies, White matter hyperintensities, Neuroimaging

## Abstract

**Purpose:**

Quantitative assessment of white matter hyperintensities (WMH) on structural Magnetic Resonance Imaging (MRI) is challenging. It is important to harmonise results from different software tools considering not only the volume but also the signal intensity. Here we propose and evaluate a metric of white matter (WM) damage that addresses this need.

**Methods:**

We obtained WMH and normal-appearing white matter (NAWM) volumes from brain structural MRI from community dwelling older individuals and stroke patients enrolled in three different studies, using two automatic methods followed by manual editing by two to four observers blind to each other. We calculated the average intensity values on brain structural fluid-attenuation inversion recovery (FLAIR) MRI for the NAWM and WMH. The white matter damage metric is calculated as the proportion of WMH in brain tissue weighted by the relative image contrast of the WMH-to-NAWM. The new metric was evaluated using tissue microstructure parameters and visual ratings of small vessel disease burden and WMH: Fazekas score for WMH burden and Prins scale for WMH change.

**Results:**

The correlation between the WM damage metric and the visual rating scores (Spearman ρ > =0.74, *p* < 0.0001) was slightly stronger than between the latter and WMH volumes (Spearman ρ > =0.72, *p* < 0.0001). The repeatability of the WM damage metric was better than WM volume (average median difference between measurements 3.26% (IQR 2.76%) and 5.88% (IQR 5.32%) respectively). The follow-up WM damage was highly related to total Prins score even when adjusted for baseline WM damage (ANCOVA, *p* < 0.0001), which was not always the case for WMH volume, as total Prins was highly associated with the change in the intense WMH volume (*p* = 0.0079, increase of 4.42 ml per unit change in total Prins, 95%CI [1.17 7.67]), but not with the change in less-intense, subtle WMH, which determined the volumetric change.

**Conclusion:**

The new metric is practical and simple to calculate. It is robust to variations in image processing methods and scanning protocols, and sensitive to subtle and severe white matter damage.

**Electronic supplementary material:**

The online version of this article (doi:10.1007/s00234-017-1892-1) contains supplementary material, which is available to authorized users.

## Introduction

White matter hyperintensities (WMH) are a common neuroradiological finding detected in T2-weighted (T2 W) and fluid attenuation inversion recovery (FLAIR) structural magnetic resonance images (MRI) in older individuals and patients with neurological diseases [1]. Their growing importance reflects the increasing recognition of their association with a wide range of disabilities [2], vascular risk factors [3] and impairments in activities of daily living [4]. Different visual rating scales are available [5–10] to assess their frequency, extent in various locations and progression [11]. However, inter- and intra-observer variation in visual assessments [12] have motivated development of computational methods for WMH burden quantification. With widespread availability of MRI technologies, there is growing expectation of incorporating these computational methods into diagnostic software.

The current literature on methods to quantify WMH burden and progression is large [13, 14]. Semi-automated or automated methods are thought to be sensitive and reproducible, but very few have been compared directly with each other, or with visual scores. Furthermore, errors in tissue classification, in exclusion or not of artefacts, and in manual correction of computer-generated masks have largely not been assessed but probably contribute to inconsistencies in results of studies of WMH associations [13]. In most cases, the computational requirements to run the most up-to-date fully automatic segmentation techniques are not specified, nor the processing time, which could be many hours for a single dataset. So far, most large clinical or population studies have used conventional histogram-based thresholding of FLAIR images to assess WMH volume (see Supplementary Table from [13]), mainly owed to its simplicity, minimal resource requirements and relative speed, and, perhaps for these reasons, there is a growing tendency to base assessment of new WMH quantification techniques on this approach. However, FLAIR-only derived thresholds are significantly affected by the WMH signal strength, anatomical distribution, extent [15] and technical factors such as bias field correction [13].

Subtle FLAIR/T2 W WMH have received special attention as they may indicate pre-lesional tissue changes [16], but are difficult to quantify as they may extend over large regions, and lack a clear boundary [13]. Thus they often appear included in “normal-appearing” white matter (NAWM). NAWM is defined as having intensities from 50 to 75% of the maximum intensity value on a T2 W-based sequence (e.g. T2 W and/or FLAIR), and are coincident with the regions classed as white matter on the human brain atlas (http://www.thehumanbrain.info/), after excluding stroke lesions and WMH [17]. When the signal strength of the WMH is high, then subtle (‘pre-lesional’) WMH could be classed as “normal” tissue and, therefore, disregarded from an evolving pathological process. Hence, it may be necessary to weight the quantitative WMH volumes (obtained from any quantification technique) by a factor that expresses the relationship between the signal strength of the regions considered WMH by the segmentation method and that of the other regions considered “normal”. This might capture the more subtle features and thus provide a more complete index of total brain injury from WMH. We propose and validate a metric to quantitatively express the white matter (WM) damage to address this need.

## Materials and methods

### Subjects

We used brain MRI data from three observational studies: a study of stroke mechanisms [18] and two studies of cognitive ageing [19, 20] (http://www.lothianbirthcohort.ed.ac.uk/). From the study of stroke, that initially enrolled 264 stroke patients (mean age 66 years (SD 11 years)), we used data from 190 patients (78 women) who had two brain MRIs at mean interval of 13 months, (SD 2 months). The median baseline stroke severity (National Institute of Health Stroke Scale score) was 2 (IQR 1–3) indicating mild stroke. From the studies of cognitive ageing we used data from: a) 38 participants of the Lothian Birth Cohort (LBC) 1921 that had an MRI scan at mean age 92 years and WMH volume measures by two different observers, one of them generated on two separate occasions, and b) 444 participants of the LBC 1936 that had an MRI scan at mean age 72.6 years (SD 0.7) and approximately three years later. Formal written consent from all subjects and ethical approval was acquired from the Lothian Research Ethics Committee (09/S1101/54, LREC/2003/2/29, REC 09/81101/54), the NHS Lothian R+D Office (2009/W/NEU/14) and the Multi-Centre Research Ethics Committee for Scotland (MREC/01/0/56) and conducted according to the principles expressed in the Declaration of Helsinki. The selection of the datasets was only based on availability of complete data required for the evaluation of the metric.

### MRI acquisition

All structural brain MRI scans were obtained at the Brain Research Imaging Centre, University of Edinburgh (http://www.bric.ed.ac.uk) on a GE Signa Horizon HDx 1.5 T clinical scanner (General Electric, Milwaukee, WI), equipped with a self-shielding gradient set and manufacturer-supplied eight-channel phased-array head coil. The imaging protocols of all three primary studies have been published previously [17, 21]. The structural sequences used in the generation of normal and abnormal WM segmentations were 3D T1-weighted and 2D axial T2 W, gradient echo and fluid attenuation inversion recovery (FLAIR) scans. Calibration sequences, magnet shimming and visual quality assurance were performed during each scanning session.

### The WM damage metric

The WM damage metric is equal to the proportion of WMH in the tissue where it can appear (i.e. brain tissue excluding the cortex), weighted by the relative contrast of the FLAIR (or T2 W) WMH with respect to that of the NAWM. If *I* is the mean FLAIR (or T2 W) signal intensity in a tissue type (e.g. in the region occupied by WMH (*I*_*WMH*_) or in the region occupied by NAWM (*I*_*NAWM*_)), these can be calculated as per Eq. 1:

1 $$ I=\frac{\sum_{i=1}^n{I}_i}{n} $$

where *n* is the total number of voxels that the tissue type occupies (i.e. WMH or NAWM) and *I*_*i*_is the FLAIR intensity of the tissue type in the voxel *i*. Then, the WM damage is:

2 $$ WMdamage=\frac{I_{WMH}-{I}_{NAWM}}{I_{NAWM}}\ast \kern.3em \frac{WMH_{volume}}{WMH_{volume}+{NAWM}_{volume}} $$

Given that the intensity level of WMH on FLAIR is conventionally reported to be at least 3 times the standard deviation (SD) above the mean intensity of the NAWM [15, 22, 23] (i.e., I_WMH_ ≥ I_NAWM_ + 3*SD_NAWM_ with 0 ≤ SD_NAWM_ ≤ (Imax – Imin)/2) (see Supplementary Fig. S1), the WM damage metric will take positive real values between 0 and 1. For minimum I_WMH_, if SD_NAWM_ = 0, I_WMH_ = I_NAWM_, meaning that WM_damage_ = 0, and if SD_NAWM_ is maximum and Imin = 0, then WM_damage_ = 3/2*(Imax/I_NAWM_)*(WMH_volume_/(WMH_volume_ + NAWM_volume_). However, for obtaining good contrast in T2 W-based sequences I_NAWM_ ≤ 2/3*Imax [24], which in the worst case scenario makes WM_damage_ dependent on the second term of the Eq. (2) (once the first term would be equal to 1) which reaches its maximum only in the hypothetical case in which all tissue is abnormal. For maximum I_WMH_, if the contrast between WMH and NAWM is maximum, the first term of Eq. (2) is also 3/2*(Imax-Imin)/I_NAWM_ (former case); and if the contrast between WMH and NAWM is minimum, the first term of the Eq. (2) is zero making WM_damage_ = 0. The WM damage metric should be defined as zero (0) in absence of WMH, i.e. when *WMH*_*volume*_ = 0.

### Image analyses

**Fig. 1:**
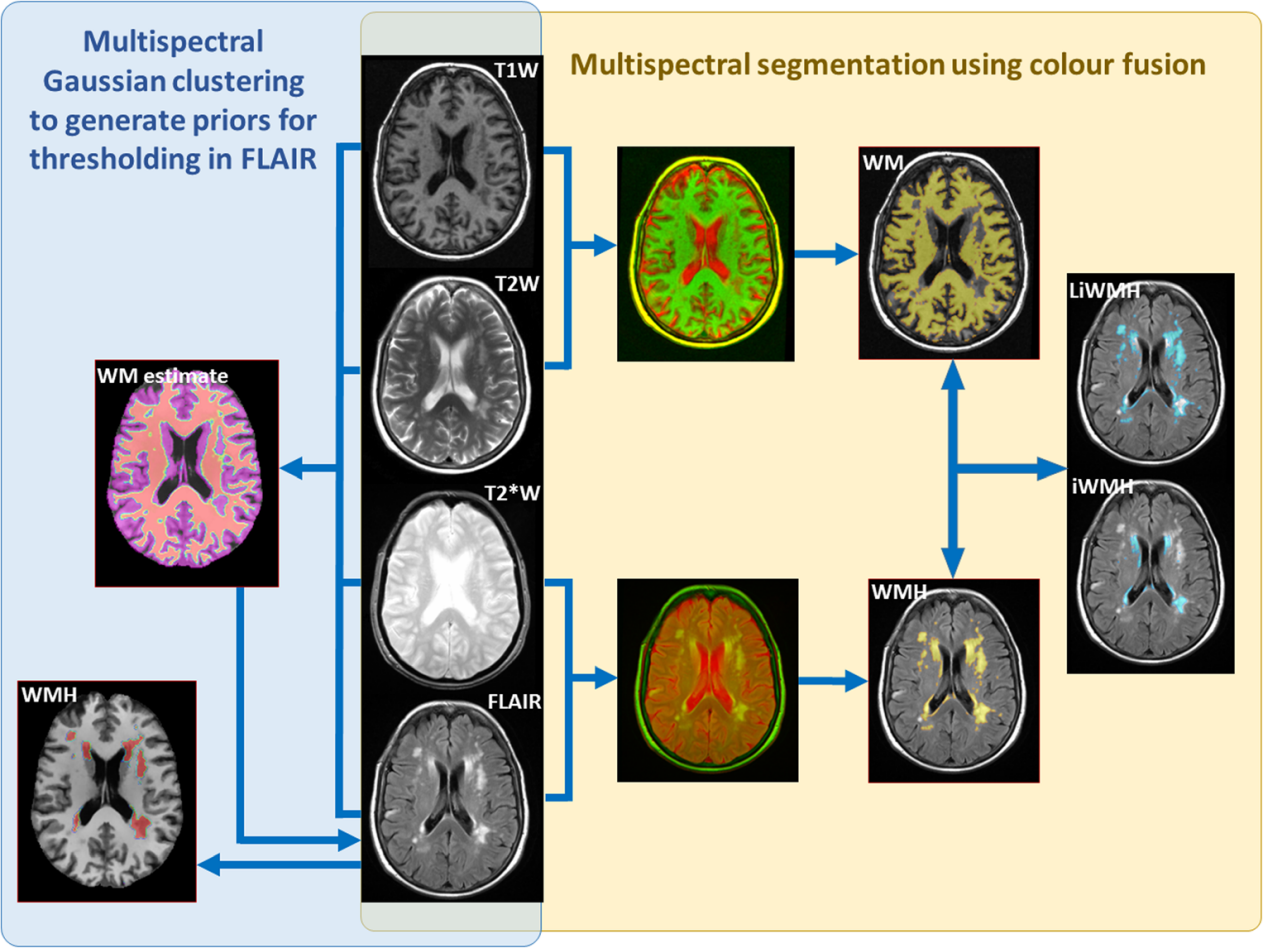
Schematic representation of the two basic NAWM and WMH segmentation methods applied to the datasets. 1) (yellow panel) Multispectral segmentation using colour fusion (MCMxxxVI, [29]) fuses in the red-green colour space T1- with T2-weighted, and T2*-weighted with FLAIR, applies minimum variance quantisation to the fused image and selects the quantised levels corresponding to WM and WMH. These are, then, combined to separate the intense from the less intense WMH and these from the normal-appearing WM. 2) (blue panel) Multispectral Gaussian clustering [26] of the multidimensional array formed by concatenating the normalised and brain-extracted FLAIR, T1-, T2- and T2*-weighted images to generate a WM likelihood estimate that generates the priors to threshold the FLAIR image and extract the WMH

We used NAWM and WMH masks obtained following the validated procedures summarised in Table 1 and schematically represented in Fig. 1. Briefly, all image sequences (from each study participant) were co-registered using FSL-FLIRT [30] and mapped to native T2 W space. WMH were extracted semi-automatically [17, 27] using a validated multispectral colour-fusion-based segmentation method (MCMxxxVI, www.sourceforge.net/projects/bric1936), which considers WMH signals that simultaneously appear in all T2 W-based sequences, and/or fully automatically using a histogram-based thresholding in the FLAIR sequence [25]. All WMH binary masks were manually edited by trained observer(s) (Table 1), who were blind to each-other’s results and to the results from the first time-point when scans from the second time-point were processed. When the multispectral colour-fusion method was applied, WMH were subdivided into intense and less-intense lesions as per [17] (Fig. 1) to analyse the influence of WMH severity on various parameters. For longitudinal analyses, the WMH volume change was calculated in two ways: 1) subtracting the numeric WMH volume at baseline from the WMH volume at follow-up and 2) subtracting previously co-registered WMH masks obtained at both time points [17]. In the samples that provided data for longitudinal analysis of WM changes, fractional anisotropy (FA) and mean diffusivity (MD) were measured in WM regions labelled as apparently normal [17] at baseline and follow-up. Stroke lesions (old and recent) were excluded [17, 27]. The WMH binary masks generated from each procedure were mapped onto the FLAIR images from which they were derived, to obtain the FLAIR intensities within each mask.

**Table 1.** Image processing methods applied to each dataset

Primary study	Number of datasets used	Evaluation	WMH segmentation method	NAWM segmentation method
Stroke	190	Longitudinal semi-automatic assessments	MCMxxxVI as per [17]. Intense and less intense regions within the WMH were separately segmented.	MCMxxxVI as per [17]
Lothian Birth Cohort (LBC) 1921	38	Automatic cross-sectional assessments and effect of inter−/intra-observer differences	1) Automatic histogram-based thresholding in FLAIR images [25] 2) Automatic histogram-based thresholding in FLAIR followed by manual editing (obs. 1, assess. 1) 3) Automatic histogram-based thresholding in FLAIR followed by manual editing (obs. 2) 4) Automatic histogram-based thresholding in FLAIR followed by manual editing (obs. 1, assess. 2)	Four-classes Multispectral Gaussian clustering [26]
Lothian Birth Cohort (LBC) 1936	237	Cross-sectional assessments from two different WMH segmentation methods	1) MCMxxxVI as per [15, 27]	1) MCMxxxVI as per [15, 27]
			2) Automatic histogram-based thresholding in FLAIR images followed by manual editing [25]	2) Four-classes Multispectral Gaussian clustering [26]
	441	Longitudinal semi-automatic assessments	Automatic histogram-based thresholding in FLAIR images followed by manual editing [25]	Probabilistic output from FSL-FAST [28]

Binary masks of NAWM were obtained using two multispectral segmentation methods (see Fig. 1): 1) MCMxxxVI by thresholding the results of applying minimum variance quantisation to the colour image resulting from fusing in the red-green colour space T1- and T2-weighted sequences (explained and validated in [29]) and/or 2) Gaussian clustering into four clusters [26] the multidimensional array formed by concatenating the normalised and brain-extracted images from FLAIR, T1-, T2- and T2*-weighted sequences. Each result was carefully visually inspected and manually edited for accuracy. Given that the multispectral Gaussian clustering did not perform well in the longitudinal ageing sample (i.e. scans taken three years apart), the binary mask of the NAWM was obtained in this sample by thresholding the probabilistic NAWM output from FSL-FAST [28]. This yielded consistent intra- and inter-subject results throughout. The rest of the output from FSL-FAST was not further used.

To evaluate the performance of our metric with respect to clinical (visual) assessments, we used visual ratings of WMH burden and WMH change, and small vessel disease (SVD) burden. WMH burden was visually rated at each time point using Fazekas [31] scores. We summed the periventricular and deep WM scores to obtain a total Fazekas score that ranged from 0 to 6.Visual ratings scores of WMH change, obtained primarily using the Prins scale [11], which assigns −1 (decrease), 0 (no change) or 1 (increase) to the perceived WMH change in three periventricular and four deep brain regions on each hemisphere, were summed to a total score that ranged from −14 to 14. Total SVD scores were obtained as per Staals et al. (2014) [32]. All visual scores were generated by a neuroradiologist with more than 25 years of experience.

### Statistical analysis

We used analysis of covariance (ANCOVA) [33, 34] with the follow-up WMH measurement as the outcome and the baseline measurement and Prins as predictors to evaluate the strength in the relation between quantitative and visual assessments of WMH change, and univariate linear regression, all programmed in SAS 9.3 (www.sas.com). For completeness, we also analysed the change scores [35] of WMH volume, Prins and WM damage.

For comparability with other existing literature [11, 27, 36] we also evaluated the bootstrapped non-parametric correlations (Spearman ρ) between the WMH volume and the burden of WM disease as assessed visually from each method and compared them with those obtained between the WM damage (i.e. the new metric) and the same visual scores. The correlations were obtained using the MATLAB Robust Correlation Toolbox (http://sourceforge.net/projects/robustcorrtool/) [37]. Paired Wilcoxon and t-tests and Bland-Altman [38] plots were used to compare the outcome of the assessments from different methods. We also calculated the bootstrapped non-parametric correlations between the WM damage and the average values of FA and MD in NAWM. We hypothesised that the FA will be lower (i.e. Spearman ρ will have negative values) and the MD will be higher (i.e. Spearman ρ will have positive values) in brains where the NAWM is not so “normal” (i.e. likely to have higher values of WM damage overall) than in brains where the NAWM is healthier.

## Results

The characteristics and parameters of each sample, as relevant to these analyses, are shown in Table 2.

**Table 2:** Characteristics of the samples involved in the evaluation of the WM damage metric

Sample \ Parameter	Longitudinal samples		Cross-sectional samples	
	Stroke	LBC1936	LBC1936 (two WMH segmentation methods)	LBC1921 (Inter−/intra-observer differences in WMH segmentation)
N (gender)	190 (112 M, 78 W)	441 (198 M, 243 W)	237 (120 M, 117 W)	38 (20 M, 18 W)
Age (years)	66 (SD 11) at baseline	72.6 (SD 0.7) at baseline	72.6 (SD 0.7)	92.1 (SD 0.34)
Total Fazekas scores baseline^†^	2 (2)	2 (1)	2 (1)	5 (3)
SVD scores baseline^†^	1 (2)	1 (2)	1(1)	2 (2)
Total Prins scores^†^	0 (4)	0 (2)	N/A	N/A
Average WMH volume (ml)^†^	Baseline scan: 12.7 (28.6) Follow-up scan: 15 (26.7)	Baseline scan: 8.1 (11.5) Follow-up scan: 11.5 (16.3)	Method 1: 9.8 (14.8) Method 2: 9.2 (12.1)	M1: 33.6 (38.1) M2: 34.9 (38.6) M3: 31.7 (41.8) M4: 32.1 (42.5)
Average % WMH vol in ICV^†^	Baseline scan: 0.86 (1.95) Follow-up scan: 0.98 (1.731)	Baseline scan: 0.57 (0.79) Follow-up scan: 0.78 (1.13)	Method 1: 0.70 (1.03) Method 2: 0.63 (0.84)	M1: 2.4 (2.5) M2: 2.5 (2.6) M3: 2.3 (2.9) M4: 2.3 (3.0)
Average WM damage^†^	Baseline scan: 0.0082 (0.025) Follow-up scan: 0.01 (0.028)	Baseline scan: 0.0065 (0.01) Follow-up scan: 0.0094 (0.016)	Method 1: 0.0075 (0.012) Method 2: 0.0073 (0.011)	M1: 0.033 (0.051) M2: 0.033 (0.051) M3: 0.032 (0.052) M4: 0.032 (0.052)

Legend: N/A: not applicable, cross-sectional data; ^†^: Values given are median (IQR); Method 1: MCMxxxVI, multispectral colour fusion-based segmentation method; Method 2: FLAIR thresholding-based segmentation method; M1-M4 refer to measurements 1 to 4 in the LBC1921 sample

## WM damage vs. WMH volume

The results from the correlations between all cross-sectional assessments are shown in Table 3 and Supplementary Table S4. In general, all correlations between cross-sectional quantitative measurements (i.e. WMH volume and WMH damage) and visual scores (i.e. total Fazekas, total SVD score) were strong and significant. WM volume and WM damage were also significantly and strongly correlated (Spearman ρ > =0.97, *p* < 0.0001) in all cases.

**Table 3:** Spearman ρ correlations (ρ; CI) between cross-sectional visual rating scores and quantitative metrics in all samples

Sample (WMH segmentation method)	Metrics	Total Fazekas	SVD score	% WMH volume in ICV	WMH volume
Stroke (MCMxxxVI) (baseline measure-ments)	Total Fazekas	**1**	0.776 [0.718 0.819]**	0.858 [0.802 0.897]**	0.852 [0.804 0.889]**
	SVD score	0.776 [0.718 0.819]**	**1**	0.740 [0.674 0.791]**	0.736 [0.664 0.790]**
	WMH volume	0.852 [0.804 0.889]**	0.736 [0.664 0.790]**	0.996 [0.994 0.997]**	1
	WM damage	0.848 [0.773 0.898]**	0.732 [0.656 0.789]**	0.987 [0.980 0.989]**	0.985 [0.978 0.989]**
LBC 1936 Study (*n* = 441) (FLAIR-based) (baseline measure-ments)	Total Fazekas	**1**	0.530 [0.451 0.602]**	0.778 [0.733 0.816]**	0.778 [0.729 0.817]**
	SVD score	0.530 [0.451 0.602]**	**1**	0.559 [0.481 0.623]**	0.558 [0.489 0.630]**
	WMH volume	0.778 [0.729 0.817]**	0.558 [0.489 0.630]**	0.994 [0.991 0.995]**	**1**
	WM damage	0.781 [0.738 0.820]**	0.556 [0.475 0.625]**	0.993 [0.990 0.994]**	0.989 [0.985 0.991]**
LBC 1936 Study (*n* = 237) (MCMxxxVI)	Total Fazekas	**1**	0.538 [0.424 0.629]**	0.728 [0.650 0.784]**	0.723 [0.641 0.782]**
	SVD score	0.538 [0.424 0.629]**	**1**	0.523 [0.419 0.613]**	0.522 [0.424 0.615]**
	WMH volume	0.723 [0.641 0.782]**	0.522 [0.424 0.615]**	0.995 [0.992 0.996]**	**1**
	WM damage	0.749 [0.674 0.807]**	0.529 [0.422 0.618]**	0.976 [0.965 0.981]**	0.972 [0.961 0.978]**
LBC 1936 Study (*n* = 237) (FLAIR-based)	Total Fazekas	**1**	0.538 [0.424 0.629]**	0.765 [0.694 0.824]**	0.763 [0.692 0.816]**
	SVD score	0.538 [0.424 0.629]**	**1**	0.530 [0.420 0.625]**	0.528 [0.419 0.626]**
	WMH volume	0.763 [0.692 0.816]**	0.528 [0.419 0.626]**	0.994 [0.991 0.995]**	**1**
	WM damage	0.767 [0.694 0.824]**	0.529 [0.428 0.627]**	0.994 [0.991 0.995]**	0.990 [0.985 0.992]**
LBC 1921 Study (average measure-ments)	Total Fazekas	**1**	0.555 [0.272 0.763]**	0.934 [0.857 0.967]**	0.944 [0.873 0.968]**
	SVD score	0.555 [0.272 0.763]**	**1**	0.602 [0.322 0.788]**	0.601 [0.318 0.779]**
	WMHvolume	0.944 [0.873 0.968]**	0.601 [0.318 0.779]**	0.983 [0.930 0.997]**	**1**
	WMdamage	0.938 [0.864 0.966]**	0.590 [0.278 0.796]**	0.989 [0.946 0.998]**	0.968 [0.907 0.987]**

Note: ** *p* < 0.0001. Correlations are shown with WM damage calculated using FLAIR images, as those using T2 W images were similar. Correlations of all the 4 measurements from the LBC1921 study sample are given in Supplementary Table S4

### WMH damage using WMH masks obtained from two different segmentation methods

The median WMH volume obtained from the two segmentation methods applied to the LBC 1936 sample of 237 individuals (samples characteristics in Tables 1 and 2) was: 9.76 ml (IQR 14.79 ml) with the multispectral method (i.e. MCMxxxVI) and 9.16 ml (IQR 12.12 ml) with the histogram-based FLAIR thresholding method. These two measurements were not statistically significantly different from each other (*p* = 0.5623). The WM damage obtained from both methods was: median value of 0.0075 for MCMxxxVI and 0.0073 for the histogram-based FLAIR thresholding method) when calculated using FLAIR images; and 0.013 for both methods when it was calculated using T2 W images. WM damage measurements did not differ significantly between methods (*p* = 0.9273). Correlations of both types of measurements (i.e. WMH volume and WM damage) with total Fazekas scores were all significant (*p* < 0.0001) regardless of whether T2 W or FLAIR signal were used in the analysis and which of the WMH segmentation methods were used (Table 3).

### Influence of inter−/ intra-observer variations in the WMH / WM damage assessments

**Fig. 2:**
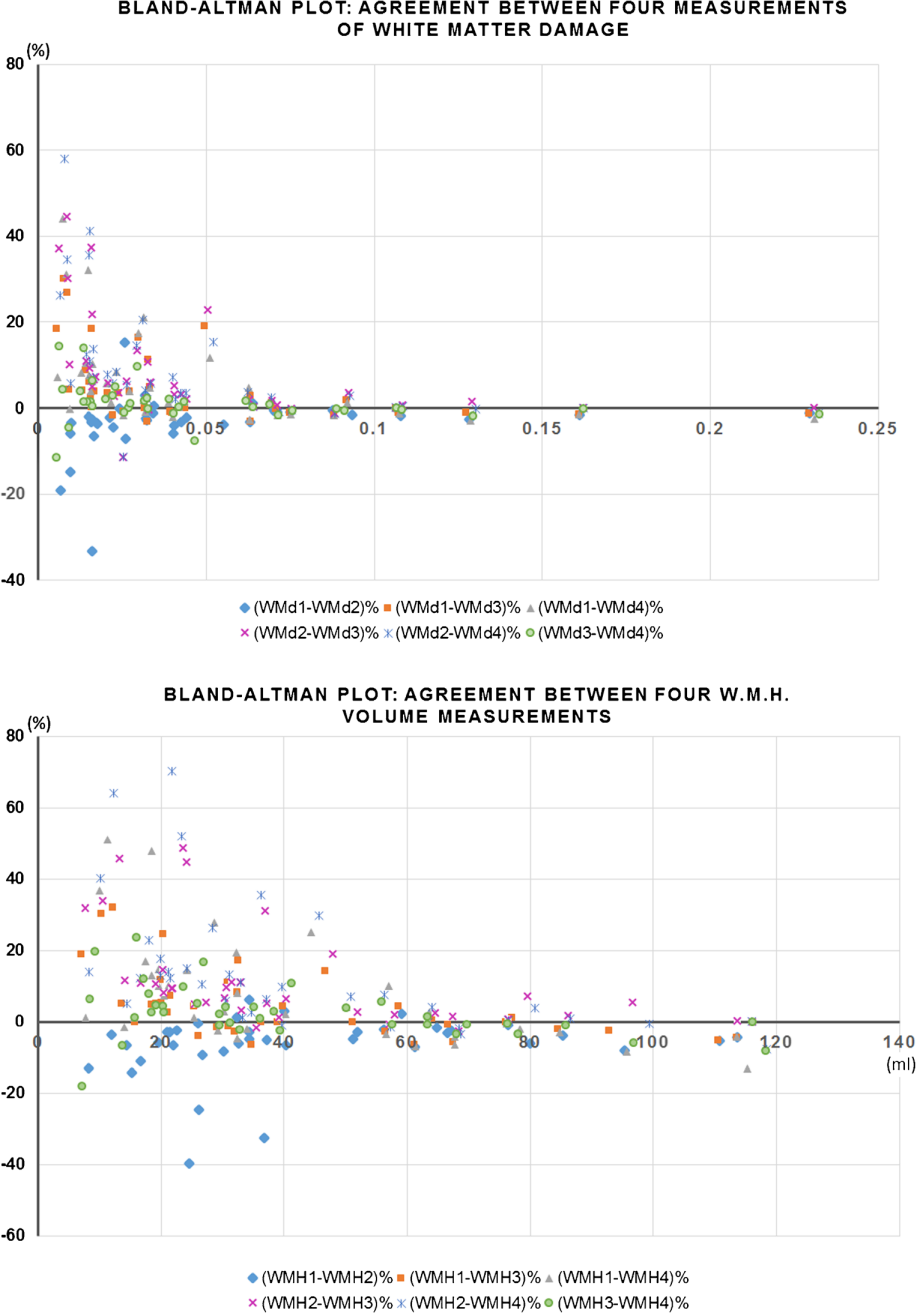
Bland-Altman plots showing the agreement between all four measurements of WM damage (above) and all four WMH volume measurements (below). Blue rhombi compare measurement 1 with 2, pink crosses compare measurements 2 and 3, orange squares evaluates the agreement of measurement 1 against 3, grey triangles compare measurements 1 and 4, green circles 3 and 4 and blue stars compare measurements 2 and 4. Ordinates of both graphs are expressed in percentage with respect to the average values for comparability of the results between both metrics. The WM damage metric shows better agreement than the WMH volume measurements, between the 4 assessments on all cases and in general (mean % differences: 4.26[−14.43 22.95](%) for the WM damage metric vs. 5.35[−18.75 29.45](%) for the WMH volume measurements)

The average median WMH volume on the ageing sample that provided data for this analysis (i.e. LBC 1921) was 33.08 ml (average IQR 40.24 ml). The average difference between the four WMH volume measurements (see Table 1) was median 5.59% (IQR 5.88%), whereas the average difference between the four WM damage metrics generated from these four assessments was median 3.26% (IQR 2.76%) when the signal intensity was assessed in the FLAIR images and 5.72% (IQR 5.32%) when the signal intensity was assessed in the T2 W images. Figure 2 and Supplementary Table S1 show the Bland-Altman analyses to evaluate agreement between all measurements involved using FLAIR images to assess the signal intensity. The WM damage metric shows better agreement than the WMH volume measurements, between the 4 assessments on all cases and in general (mean % differences: 4.26[−14.43 22.95](%) for the WM damage metric vs. 5.35[−18.75 29.45](%) for the WMH volume measurements). Similar results were observed using T2 W to quantify the signal intensity. Figure S2 (Supplementary material) shows the four measurements of WMH volume and WM damage for all the 38 datasets of this sample using FLAIR images to assess the signal intensity. Non-parametric correlations (Spearman ρ) between the WM damage metric and visual rating scores were all significant (*p* < 0.001) and slightly stronger than between WMH volume and visual ratings (Table 3 and Supplementary Table S4).

## WM damage progression vs. longitudinal WMH change

The net volume of WMH increased (median 2.6 ml (IQR 4.7 ml)) over 3 years in the ageing sample, and at approximately 1 year in the stroke sample (median 1.4 ml (IQR 6.9 ml)) (baseline and follow-up volumes in Table 2). The total Prins visual ratings ranged from −12 to +10 in stroke patients, and correlated significantly with the progression of WM damage expressed by our new metric (Spearman ρ between WM damage change and Total Prins = 0.5, *p* < 0.0001, for both samples) (Tables S2 and S3). In both samples, the follow-up WM damage was highly related to total Prins score even when adjusted for baseline WM damage (ANCOVA, *p* < 0.0001): the increase in the logarithm of WM damage per unit change in total Prins score was 0.074 (95%CI [0.054 0.093]) in the stroke sample, and 8.67 × 10^−4^ (95%CI [8.05 × 10^−4^9.30 × 10^−4^]) in the ageing sample. However, in the stroke sample total Prins score and WMH volume change (calculated by subtracting the WMH volumes at both time points) were not correlated (Table S2).

**Fig. 3:**
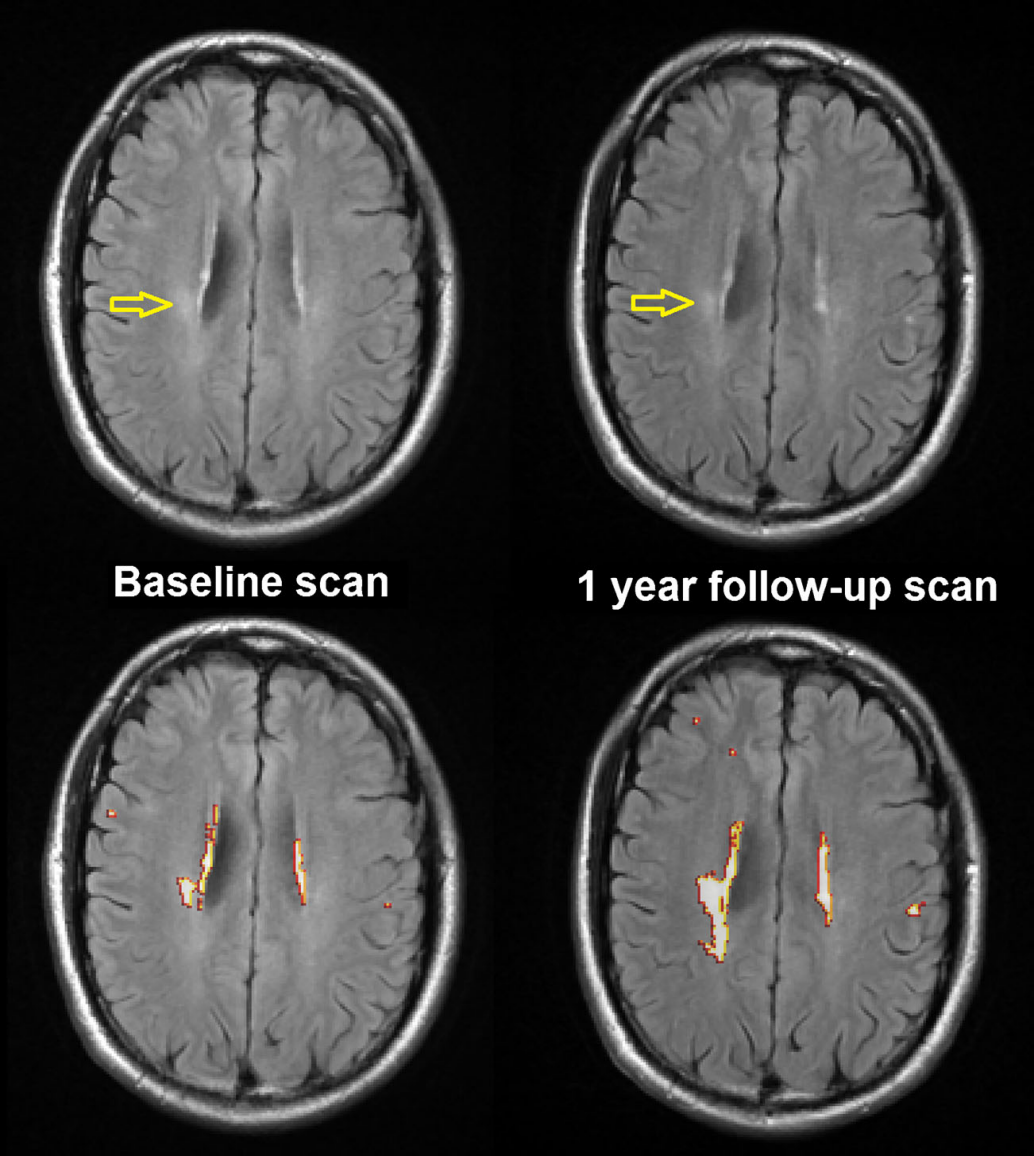
Axial FLAIR slice of the baseline and follow-up scans of a stroke patient, in which the total WMH volume changed from 4.6 ml to 11 ml and the WM damage metric changed from 0.0036 to 0.0058. The intense WMH increased 0.7 ml (i.e. from 2 to 2.7 ml), whereas the less intense WMH (arrowed) increased 5.7 ml (i.e. from 2.6 to 8.3 ml). Despite both images been acquired with equal scanning parameters, the contrast between the subtle WMH and the “normal” WM differs in both scans influencing the results of the segmentation. Below are shown the WMH masks overlaid in both scans

**Fig. 4:**
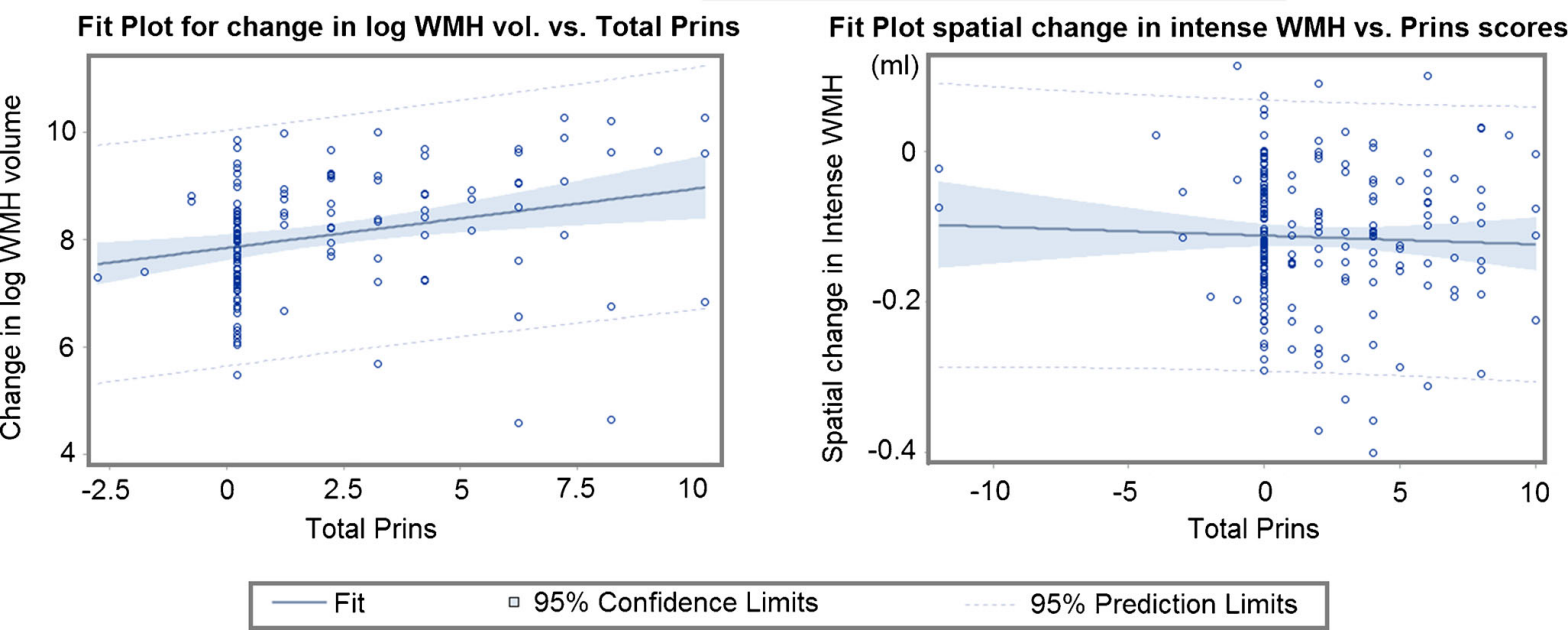
The change in WMH volume, after being log transformed, was modestly but significantly associated with Total Prins (*left*) in the stroke sample. However, the spatial change (obtained from the subtraction of the baseline and follow-up WMH masks, not from the subtraction of the volumetric measurements) observed in the regions of intense WMH [17], was not associated with Total Prins (*right*)

Further analysis of WMH severity in the stroke patients revealed that the net WMH volume change was mainly driven by the change in the volume of less intense WMH (Fig. 3) (i.e. the correlation between net WMH volume change and the change in the less intense WMH was stronger (Spearman ρ 0.90, *p* < 0.0001) than with the change in the intense WMH (Spearman ρ 0.46, *p* < 0.0001)). On the contrary, total Prins scores were more closely related to the change in the volume of the intense WMH (Table S2). Indeed, the follow-up intense WMH volume was significantly associated to total Prins score (*p* = 0.0079), with an increase of 4.42 ml per unit change in total Prins score (95%CI [1.17 7.67]) after adjusting for baseline intense WMH volume. An univariate regression analysis of the spatial change in WMH (i.e. obtained from the subtraction of the baseline and follow-up WMH masks, not from the subtraction of the volumetric measurements) in relation to Total Prins in this sample showed a logarithm of the total change in WMH increasing in 0.11 per unit change in Total Prins, (95%CI [0.041 0.18], *p* = 0.0019) and no association between the change of the intense WMH and Total Prins (*p* = 0.57) (Fig. 4).

## WM damage and WM microstructure (FA, MD)

In the NAWM regions, WM damage was negatively correlated with average values of FA (Spearman ρ ≈ −0.4 for the LBC1936 sample and ≈ −0.55 for the stroke sample) and positively correlated with average values of MD (Spearman ρ ≈ 0.3 for the LBC1936 sample and ≈ 0.6 for the stroke sample) in all cases at baseline and follow-up, (all correlations *p* < 0.0001).

## Analysis of possible confounds

All evaluations were repeated considering the WMH volumes before and after correcting for head size (Table 3 and Supplementary Tables S2 to S4). Paired non-parametric Wilcoxon and t-tests showed that despite the median WMH volume before and after adjusting by head size (i.e. intracranial volume (ICV)) being significantly different from each other (*p* < 0.0001), and the percentage change of WMH in ICV being significantly different from the percentage change of WMH volume (*p* < 0.0001) on all samples evaluated, the strength and significance of all correlations and associations were unaltered (see cells in grey in Supplementary Tables 2 and 3), and their correlation was 0.998 [0.997 0.999] in the stroke sample and 0.996 [0.994 0.997] in the ageing sample, significant in both samples (*p* < 0.0001).

Analyses were repeated using the WM damage calculated using T2 W images. In measurements labelled as 2 (M2 in Table 2) in the LBC1921 sample, the WM damage calculated using FLAIR differed from when it was calculated using T2 W with borderline significance (*p* = 0.047), but in general, this difference was not significant (*p* > 0.05). The strength in the Spearman correlations between the WM damage metric calculated from T2 W or FLAIR and the visual ratings (i.e. Fazekas and SVD scores) was the same up to the second decimal point for large samples, and differed in the absolute range of 0.02 in the small sample (LBC 1921, *n* = 38) except when the WM damage obtained from T2 W and FLAIR differed with borderline significance (Spearman ρ correlations with visual ratings differed in 0.05) (see Supplementary Table S5). The significance in the correlations was not altered.

## Discussion

There is a need to harmonise methods for processing common features such as WMH on imaging. We present a WM damage metric that expressed the degree of WM disease on conventional structural MRI and was robust to variations in image processing methods used to quantify “normal” and “abnormal” WM. Although we have focused here on WMH of presumed vascular origin, this metric could be used to harmonise results in studies of other neurological diseases that present WMH on MRI. Once NAWM and WMH regions are defined on brain MRI, the WM damage metric is very simple to compute. Only T2 W and FLAIR have been tested and proposed for generating this metric because WM disease is identified on structural MRI by these two sequences, specifically WMH are defined as showing “hyperintensity on T2-weighted images such as fluid-attenuated inversion recovery” [1]. The value of the WM damage metric is not absolute. It depends on the type and parameters of the sequence used (i.e. T2 W or FLAIR), as it is calculated using intensity (i.e. not quantitative) images. However, the associations between WMH burden using this parameter and neuroradiological assessments of WMH burden and WMH change may remain invariant despite variations in the scanning protocols and/or sequence used to compute it. Given the variety of sequences and scanning parameters from the several MRI scanner manufacturers, this fact gives additional strength.

Whilst the correlations between the new metric and visually assessed WMH burden (i.e. Fazekas scores) are comparable with those between the WMH volumetric measurements and the visual scores, the results suggest that the change (i.e. longitudinal change, evolution) in WMH burden is better and more consistently represented by this metric than by conventional volumetric methods. Of particular interest is the consistency in the associations between the neuroradiological visual assessments of longitudinal WMH changes (i.e. Prins visual scores) and the difference in the WM damage metric (i.e. WM damage change). On the other hand, assessments of WMH volume change are not always straightforward and therefore volumetric and visual assessments of WMH change do not always agree. Depending on whether the WMH volume change is calculated, e.g. by subtracting the WMH volumes obtained at different time points or analysing the spatial change, its association with the visual scores can differ, as in the longitudinal samples we analysed. Analysing the spatial change of the regions labelled as WMH would be the most reasonable approach, but image co-registration in the presence of tissue loss and shape distortions in the brain parenchyma (e.g. brain images of stroke patients) can be challenging and misleading.

The robustness of the new metric to differences in image processing methods and its sensitivity to capture subtle and severe white matter damage confer it advantage over the use of WMH volume in the assessment of longitudinal WM changes. In addition, analysis of tissue microstructure in regions labelled as “normal” showed agreement with the degree of WM damage expressed by the proposed metric. We think that using this metric together with the conventional indicators of disease currently available will be a step forward in the harmonisation of study results, computational diagnostics and “big data” analyses. Further evaluation using images from other diseases (e.g. multiple sclerosis, different types of dementia, etc.) is needed.

## Electronic supplementary material

Figure S1 (GIF 491 kb).

High resolution image (TIFF 519 kb).

Figure S2 (PNG 99 kb).

ESM 1 (DOCX 20 kb).

## Abbreviations

WMH: White matter hyperintensities
FLAIR: Fluid Attenuated Inversion Recovery
MRI: Magnetic resonance imaging
NAWM: Normal-appearing white matter
CI: Confidence interval
IQR: Interquartile range
