# Supplementary figures and images for: Metric to quantify white matter damage on brain magnetic resonance images
Source: Neuroradiology. 2017 Aug 16;59(10):951–62. doi: 10.1007/s00234-017-1892-1 (PMC5596039; doi:10.1007/s00234-017-1892-1)

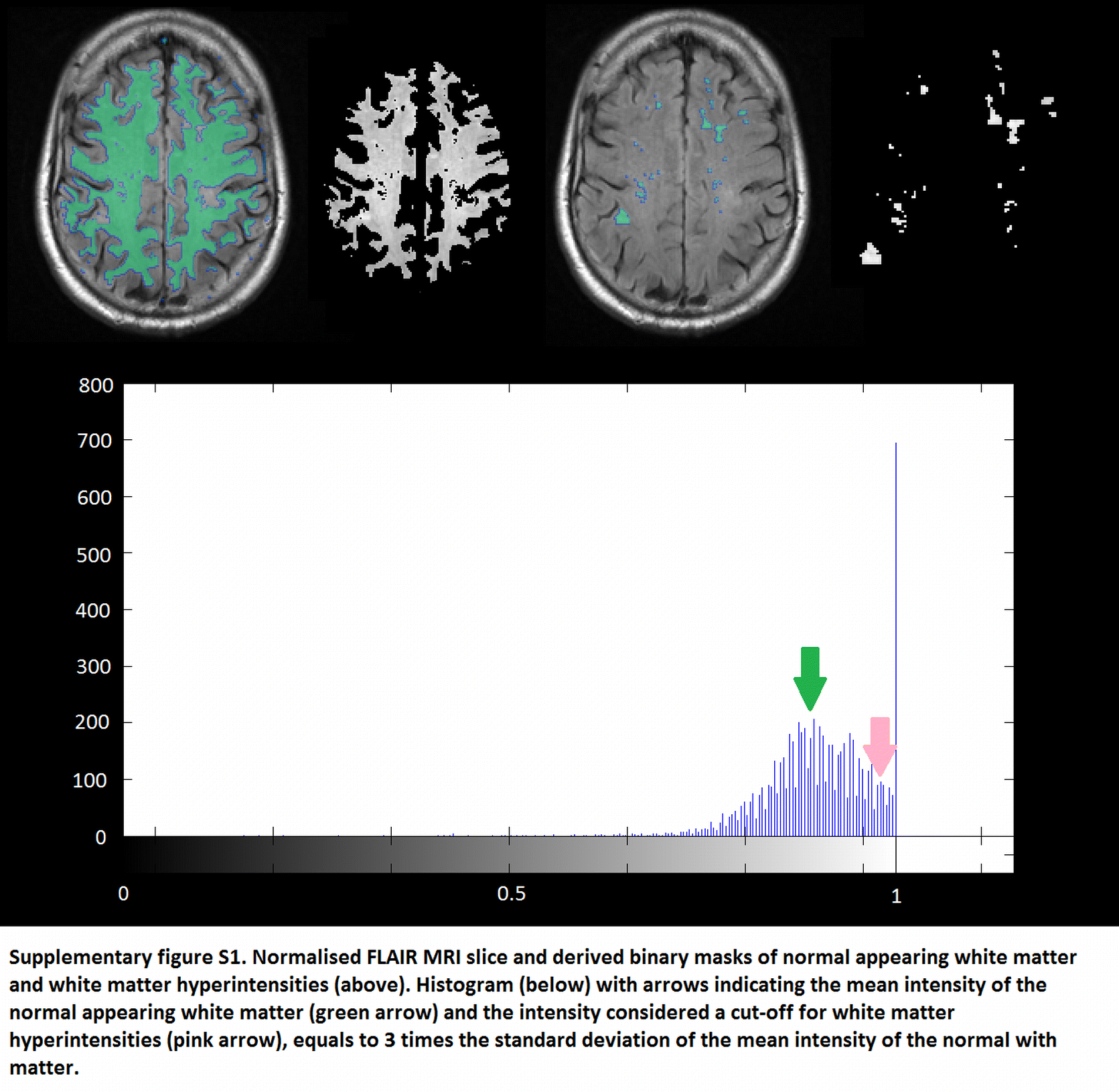

Supplement: Supplementary file 1 — (GIF 491 kb). [file 234_2017_1892_Fig5_ESM.gif]

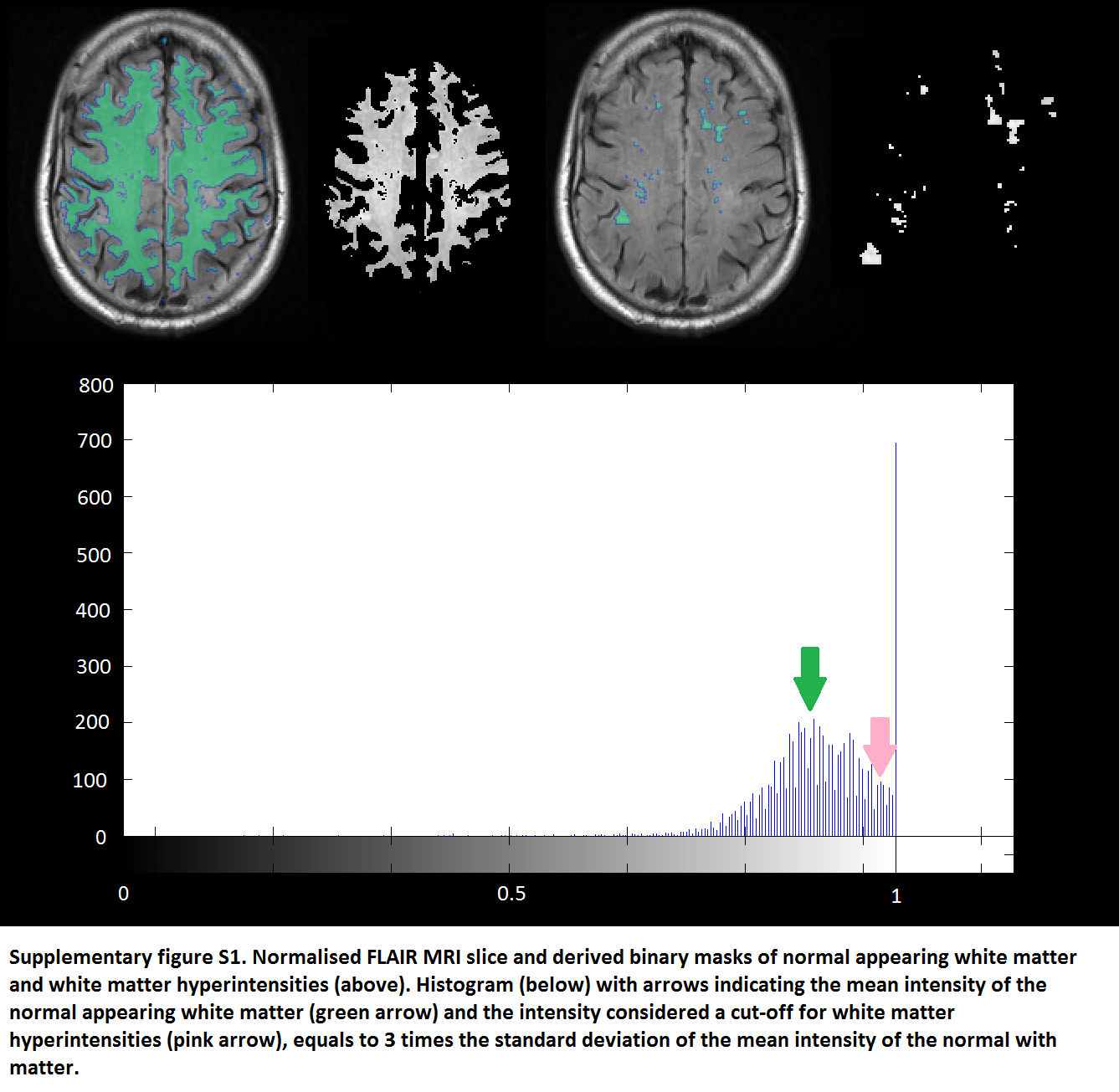

Supplement: Supplementary file 2 — High resolution image (TIFF 519 kb). [file 234_2017_1892_MOESM1_ESM.tif]

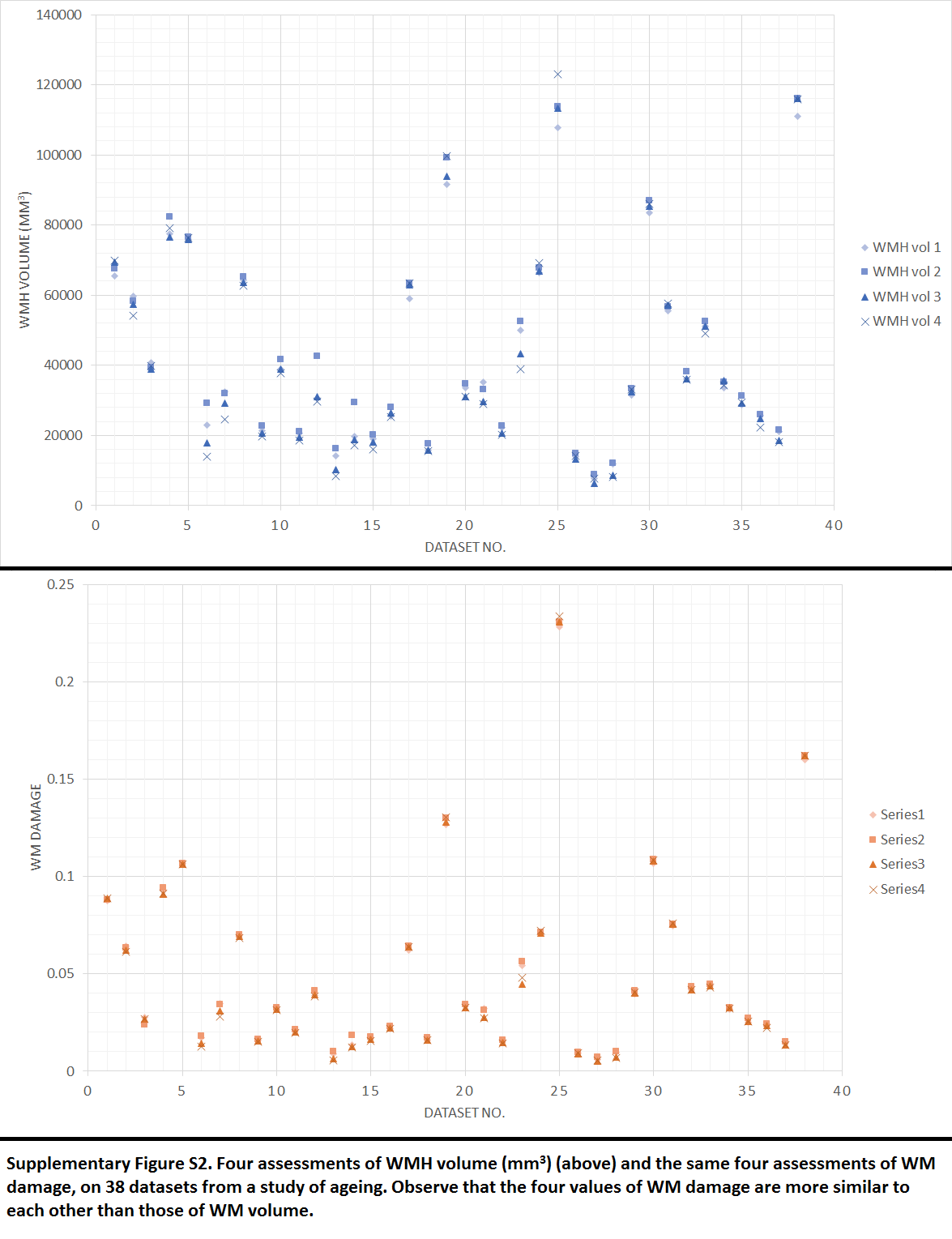

Supplement: Supplementary file 3 — (PNG 99 kb). [file 234_2017_1892_MOESM2_ESM.png]
