# Supplementary material for: Metric to quantify white matter damage on brain magnetic resonance images
Source: Neuroradiology. 2017 Aug 16;59(10):951–62. doi: 10.1007/s00234-017-1892-1 (PMC5596039; doi:10.1007/s00234-017-1892-1)
Supplement: Supplementary file 4 — (DOCX 20 kb). [file 234_2017_1892_MOESM3_ESM.docx]

**Metric to quantify white matter damage on brain magnetic resonance images**

**Supplementary Material**

Table S1. Bland-Altman analysis of inter-/intra-observer agreements between four assessments of white matter damage and volumetric measurements of white matter hyperintensities (LBC1921 sample). Given: Difference [-95%CI +95%CI].

| Measurements no. | Absolute values | | Differences relative to the average values between both measurements (%) | |
| --- | --- | --- | --- | --- |
|  | White matter damage  (x10^-3^) | Volumes of white matter hyperintensities (ml) | White matter damage | Volumes of white matter hyperintensities |
| 1 and 2 | -0.9 [-3.77 1.85] | -2.36 [-8.17 3.44] | -3.47 [-17.44 10.51] | -6.57 [-24.20 11.07] |
| 1 and 3 | 0.67 [-3.56 4.92] | 0.45 [-4.94 5.84] | 4.60 [-11.97 21.16] | 4.44 [-14.82 23.70] |
| 1 and 4 | 0.73 [-4.11 5.58] | 0.72 [-8.63 10.07] | 5.85 [-15.35 27.05] | 7.07 [-22.15 36.29] |
| 2 and 3 | 1.64 [-2.84 6.11] | 2.81 [-3.57 9.20] | 8.03 [-15.85 31.92] | 10.96 [-16.28 38.19] |
| 2 and 4 | 1.70 [-2.89 6.28] | 3.09 [-6.09 12.26] | 9.28 [-18.00 36.56] | 13.53 [-22.54 49.58] |
| 3 and 4 | 0.05 [-2.24 2.35] | 0.27 [-4.76 5.30] | 1.27 [-7.98 10.53] | 2.68 [-12.52 17.88] |

*Legend: SD: standard deviation; CI: confidence intervals*

Table S2. Spearman ρ correlations between Prins visual rating scores and quantitative assessments of net WM change in the stroke sample (n=190), exploring WMH type (periventricular (PV) or patches in the deep white matter) and severity (intense and less intense WMH). Given (ρ [confidence interval])

|  | Total PV Prins | Total deep Prins | Total Prins | Total WMH volume change | WM damage change |
| --- | --- | --- | --- | --- | --- |
| Total deep Prins | **0.571 [0.448 0.673]**** | 1 |  |  |  |
| Total Prins | **0.808 [0.745 0.858]**** | **0.921 [0.881 0.952]**** | 1 |  |  |
| Total WMH volume change | 0.016 [-0.129 0.187] | 0.112 [-0.052 0.267] | 0.069 [-0.092 0.226] | 1 |  |
| WM damage change | **0.412 [0.262 0.542]**** | **0.401 [0.253 0.535]**** | **0.455 [0.307 0.572]**** | **0.598 [0.464 0.719]**** | 1 |
| % WMH volume change in ICV | 0.014 [-0.157 0.169] | 0.108 [-0.048 0.263] | 0.065 [-0.098 0.225] | **0.998 [0.997 0.999]**** | **0.595 [0.454 0.713]**** |
| Intense WMH volume change | **0.243 [0.060 0.388]**** | **0.314 [0.157 0.463]**** | **0.324 [0.168 0.478]**** | **0.463 [0.328 0.588]**** | n/a |
| Less intense WMH volume change | -0.0007 [-0.164 0.168] | 0.0275 [-0.140 0.193] | 0.0032 [-0.159 0.165] | **0.895 [0.838 0.940]**** | n/a |

Note: The significance in the correlations did not change when the outliers were excluded, although they became stronger, ** p<0.0001

n/a: not applicable. The white matter damage was not determined between normal-appearing white matter and regions of intense (i.e. severe) and less intense (i.e. less severe) WMH separately as it expresses, *per se*, the degree of health, severe and less severe damage of the white matter.

ICV : Intracranial volume

The volumetric change was calculated as the subtraction of the volumes obtained at baseline from those obtained at follow-up.

Cells in gray highlight the values of the correlations obtained before and after adjusting the WMH volumes for head size, for facilitating their comparison.

Table S3. Spearman ρ correlations between Total Prins visual rating scores and quantitative assessments of WM change in the LBC1936 ageing sample (n=441)

|  | Total Prins | Total % WMH volume change in ICV | Total WMH volume change | WM damage change |
| --- | --- | --- | --- | --- |
| Total Prins | 1 | **0.517 [0.435 0.591]**** |  |  |
| Total WMH volume change | **0.512 [0.432 0.584]**** | **0.996 [0.994 0.997]**** | 1 |  |
| WM damage change | **0.501 [0.419 0.571]**** | **0.979 [0.967 0.985]**** | **0.972 [0.961 0.980]**** | 1 |

Note: The significance in the correlations did not change when the outliers were excluded, although they became stronger, ** p<0.0001

Cells in gray highlight the values of the correlations obtained before and after adjusting the WMH volumes for head size, for facilitating their comparison.

Table S4. Spearman ρ correlations (ρ; CI) between cross-sectional visual rating scores and all four quantitative metrics of white matter hyperintensity (WMH) volume, percentage of WMH in intracranial volume, and white matter damage, from the LBC1921 sample. (**: p<0.0001)

| Measurement no. | Metrics | Total Fazekas | SVD score | % WMH volume in ICV | WMH volume |
| --- | --- | --- | --- | --- | --- |
| 1 | Total Fazekas | 1 | 0.55 [0.274 0.747]** | 0.934 [0.859 0.968]** | 0.939 [0.862 0.969]** |
|  | SVD score | 0.55 [0.274 0.747]** | 1 | 0.601 [0.311 0.804]** | 0.581 [0.249 0.783]** |
|  | WMH volume | 0.939 [0.862 0.969]** | 0.581 [0.249 0.783]** | 0.980 [0.938 0.992]** | 1 |
|  | WMH damage | 0.930 [0.843 0.966]** | 0.594 [0.290 0.810]** | 0.984 [0.946 0.994]** | 0.966 [0.906 0.986]** |
| 2 | Total Fazekas | 1 | 0.55 [0.274 0.747]** | 0.930 [0.841 0.964]** | 0.940 [0.874 0.967]** |
|  | SVD score | 0.55 [0.274 0.747]** | 1 | 0.639 [0.398 0.813]** | 0.618 [0.329 0.783]** |
|  | WMH volume | 0.940 [0.874 0.967]** | 0.618 [0.329 0.783]** | 0.982 [0.949 0.993]** | 1 |
|  | WMH damage | 0.931 [0.851 0.963]** | 0.624 [0.366 0.796]** | 0.984 [0.952 0.994]** | 0.970 [0.920 0.987]** |
| 3 | Total Fazekas | 1 | 0.55 [0.274 0.747]** | 0.941 [0.871 0.971]** | 0.949 [0.887 0.973]** |
|  | SVD score | 0.55 [0.274 0.747]** | 1 | 0.578 [0.282 0.792]** | 0.578 [0.313 0.777]** |
|  | WMH volume | 0.949 [0.887 0.973]** | 0.578 [0.313 0.777]** | 0.983 [0.951 0.994]** | 1 |
|  | WMH damage | 0.938 [0.865 0.967]** | 0.582 [0.277 0.798]** | 0.989 [0.962 0.996]** | 0.975 [0.928 0.990]** |
| 4 | Total Fazekas | 1 | 0.55 [0.274 0.747]** | 0.942 [0.866 0.969]** | 0.949 [0.892 0.972]** |
|  | SVD score | 0.55 [0.274 0.747]** | 1 | 0.579 [0.292 0.794]** | 0.588 [0.310 0.781]** |
|  | WMH volume | 0.949 [0.892 0.972]** | 0.588 [0.310 0.781]** | 0.985 [0.950 0.994]** | 1 |
|  | WMH damage | 0.938 [0.867 0.968]** | 0.589 [0.270 0.796]** | 0.985 [0.950 0.994]** | 0.971 [0.913 0.989]** |

Table S5. Spearman ρ correlations (ρ; CI) between cross-sectional visual rating scores and all white matter damage values obtained from the LBC1921 sample and calculated using the intensity values of T2W on the WMH and NAWM regions vs. those obtained using FLAIR. (**: p<0.0001)

| Sequence | Measurement | Total Fazekas | SVD score |
| --- | --- | --- | --- |
| T2W | 1 | 0.907 [0.806 0.958]** | 0.595 [0.290 0.792]** |
|  | 2 | 0.879 [0.763 0.934]** | 0.632 [0.358 0.804]** |
|  | 3 | 0.918 [0.812 0.960]** | 0.594 [0.307 0.807]** |
|  | 4 | 0.920 [0.820 0.966]** | 0.610 [0.315 0.808]** |
| FLAIR | 1 | 0.930 [0.850 0.966]** | 0.594 [0.270 0.800]** |
|  | 2 | 0.930 [0.845 0.965]** | 0.624 [0.348 0.789]** |
|  | 3 | 0.938 [0.868 0.968]** | 0.582 [0.281 0.792]** |
|  | 4 | 0.938 [0.866 0.968]** | 0.589 [0.289 0.792]** |
